# A Rasch Model and Rating System for Continuous Responses Collected in Large-Scale Learning Systems

**DOI:** 10.3389/fpsyg.2020.500039

**Published:** 2020-12-18

**Authors:** Benjamin Deonovic, Maria Bolsinova, Timo Bechger, Gunter Maris

**Affiliations:** ^1^ACT, Inc., Iowa City, IA, United States; ^2^Department of Methodology and Statistics, Tilburg University, Tilburg, Netherlands; ^3^ACT, Inc., Amsterdam, Netherlands; ^4^Department of Psychological Methods, University of Amsterdam, Amsterdam, Netherlands

**Keywords:** Rasch model, longitudinal data analysis, rating system, item response theory (IRT), learning and assessment system, continuous response measurement

## Abstract

An extension to a rating system for tracking the evolution of parameters over time using continuous variables is introduced. The proposed rating system assumes a distribution for the continuous responses, which is agnostic to the origin of the continuous scores and thus can be used for applications as varied as continuous scores obtained from language testing to scores derived from accuracy and response time from elementary arithmetic learning systems. Large-scale, high-stakes, online, anywhere anytime learning and testing inherently comes with a number of unique problems that require new psychometric solutions. These include (1) the cold start problem, (2) problem of change, and (3) the problem of personalization and adaptation. We outline how our proposed method addresses each of these problems. Three simulations are carried out to demonstrate the utility of the proposed rating system.

## 1. Introduction

Large-scale, high-stakes, online, anywhere anytime learning and testing inherently comes with a number of unique problems that require new psychometric solutions. First, there is the *cold start problem*: the system needs to start without data. The traditional solution is to start with a large item bank calibrated to an appropriate *Item Response Theory (IRT) model*, which is expensive and challenging as it requires large numbers of representative test takers to respond to items under realistic testing conditions. Second, there is the *problem of change*: learner and item properties change as a cohort of learners progresses through its education. While such changes are intended, they are not easily handled by traditional psychometrics developed to assess student's ability at a single time point. Finally, there is *the problem of personalization and adaptation*: to optimally support learning, each learner follows her own path at her own pace. This will give rise to sparse, incomplete data that are not easily analyzed using likelihood-based methods. Moreover, online learning systems, such as Duolingo, for foreign languages, and Math Garden, for elementary arithmetic, generate large data sets with large number of item responses per learner as learners practice with many items over extended periods of time.

The urnings rating system was introduced by Bolsinova et al. (2020) to address these challenges, but its usefulness is limited by the fact that it assumes a Rasch model (or its generalization for polytomous data) and is tied to discrete item responses. In this paper, we extend the urnings rating system to continuous responses and illustrate its relevance for online learning systems using simulated data. Throughout, the Duolingo English Test (DET; Wagner and Kunnan, 2015; LaFlair and Settles, 2019; Maris, 2020), and Math Garden (Klinkenberg et al., 2011) will serve as motivating examples.

## 2. The Continuous Rasch Model

Continuous responses can be obtained from a wide variety of data and functions of data. In the DET, item responses are continuous numbers between zero and one. In Math Garden, continuous responses come from a combination of accuracy and time. Other learning and assessment systems may ask users to provide their perceived certainty that the chosen response is correct (Finetti, 1965; Dirkzwager, 2003). In this paragraph, we consider a general measurement model for continuous responses. For expository purposes, we consider the responses to be between zero and one.

**Figure 1:**
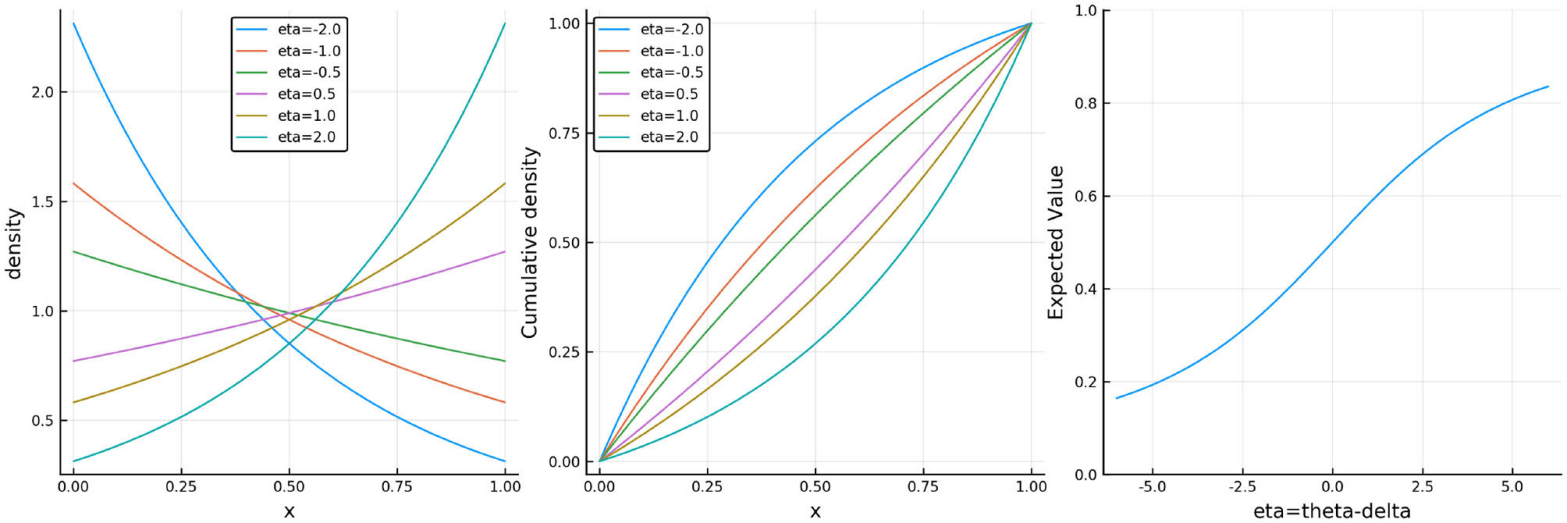
(Left) The probability density function, (middle) the cumulative distribution function, and (right) the expectation of the continuous Rasch model where η = θ − δ_*i*_.

The model we consider is the direct extension of the Rasch model to continuous responses and we will refer it as *the continuous Rasch (CR) model*. Suppressing the person index, the CR model is defined by the following response probabilities:

(1) $$\begin{array}{rc} f\left(\text{x}|\theta \right) & =\underset{i}{\prod }f\left(x_{i}|\theta \right) \end{array}$$

(2) $$\begin{array}{r} \;=\underset{i}{\prod }\frac{\exp\left(x_{i}\left(\theta -\delta_{i}\right)\right)}{\int_{0}^{1}\exp\left(s\left(\theta -\delta_{i}\right)\right)ds} \end{array}$$

(3) $$\begin{array}{r} \;=\underset{i}{\prod }\frac{\left(\theta -\delta_{i}\right)\exp\left(x_{i}\left(\theta -\delta_{i}\right)\right)}{\exp\left(\theta -\delta_{i}\right)-1}, \end{array}$$

where θ represents learner ability and δ_*i*_ item difficulty. This is an exponential family IRT model where the sum $x_{+}=\underset{i}{\sum }x_{i}$ is the sufficient statistic for ability. Note that the CR model is not new as it is equivalent^1^ to the Signed Residual Time (SRT) model proposed by Maris and van der Maas (2012) and the Rasch model for continuous responses found in Verhelst (2019). The key insight is that the model can be used for any type of continuous responses. For illustration, Figure 1 shows plots of the probability density, cumulative distribution, and expectation functions under the CR model.

For our present purpose, we will not analyze the continuous responses directly but a limited number of binary responses derived from them. We now explain how this works. If we define two new variables as follows

(4) $$\begin{array}{r} y_{i1}=\left(x_{i}>0.5\right) \end{array}$$

(5) $$\begin{array}{r} x_{i1}=\left\{ \begin{array}{cc} x_{i}-0.5 & \text{if}y_{i1}=1\\ x_{i} & \text{if}y_{i1}=0 \end{array}\right. \end{array}$$

we obtain conditionally independent sources of information on ability from which the original observations can be reconstructed; that is, *Y*_*i*1_⊥  ⊥*X*_*i*1_|θ. Moreover, it is readily found that the implied measurement model for *Y*_*i*1_ is the Rasch model:

(6) $$\begin{array}{r} p\left(Y_{i1}=1|\theta \right)=p\left(X_{i}>0.5|\theta \right)=\frac{\exp\left(0.5\left(\theta -\delta_{i}\right)\right)}{1+\exp\left(0.5\left(\theta -\delta_{i}\right)\right)} \end{array}$$

where the discrimination is equal to a half. The other variable, *X*_*i*1_, is continuous with the following distribution over the interval 0 to 1/2:

(7) $$\begin{array}{r} f\left(x_{i1}|\theta \right)=\frac{\left(\theta -\delta_{i}\right)\exp\left(x_{i1}\left(\theta -\delta_{i}\right)\right)}{\exp\left(0.5\left(\theta -\delta_{i}\right)\right)-1} \end{array}$$

The distribution of *X*_*i*1_ and *X*_*i*_ thus belong to the same family, but with a different range for the values of the random variable. We can now continue to split up *X*_*i*1_ into two new variables and recursively transform the continuous response to a set of conditionally independent Rasch response variables with discriminations that halve in every step of the recursion.

**Figure 2:**
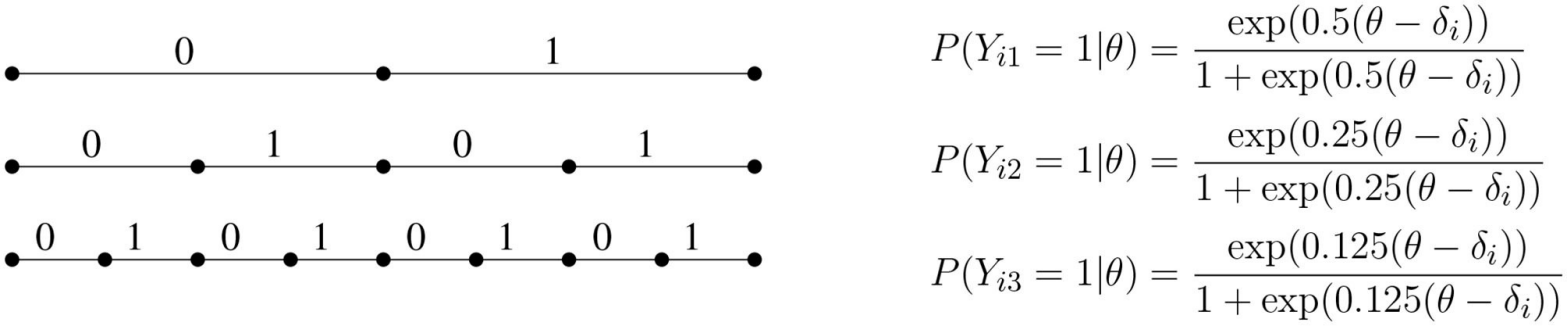
The first three steps of a dyadic expansion of continuous responses into conditionally independent binary response variables. Each follows a Rasch model with a discrimination that halves at each subsequent step.

If we denote the binary response variable obtained in the *j*-th step of the recursion by *Y*_*ij*_, we obtain the (non-terminating) dyadic expansion (see e.g., Billingsley, 2013) of the continuous response variables into conditionally independent binary response variables, as depicted in Figure 2. Since the discriminations halve in every step, most of the statistical information about ability contained in the continuous response is recovered by a limited number of binary variables. If the CR model fits, then at the point where θ = δ_*i*_ the information in the continuous response is $\frac{4}{3}$ times the information contained in *Y*_*i*1_ alone^2^.

Other models have been developed for continuous responses. Notably the extensions by Samejima to the graded response models (Samejima, 1973, 1974), Müller's extension to Andrich's rating formulation (Müller, 1987), and more recently, a generalization of the SRT model (van Rijn and Ali, 2017). Estimation procedures developed for these models have all been likelihood based and quite infeasible in a learning setting where there are many people and items, and each person answers a different subset of items. For the CR model, we will therefore turn to estimation via the use of rating systems.

## 3. Methods: The Urnings Rating System

### 3.1. Classic Urnings

Adaptive online tests produce data sets with both a large number of test takers and a large number of items. Even when we analyze binary response variables, direct likelihood-based inference will not scale-up to handle these large amounts of data. We will therefore use a rating system. A rating system is a method to assess a player's strength in games of skill and track its evolution over time. Here, learners solving items are considered players competing against each other and the ratings represent the skill of the learner and the difficulty of the item.

Rating systems, such as the Elo rating system (Elo, 1978; Klinkenberg et al., 2011), originally developed for tracking ability in chess, are highly scalable but come with their own set of problems. Elo ratings, in particular, are known to have an inflated variance, and their statistical properties are not very well-understood (e.g., Brinkhuis and Maris, 2009). The urnings rating system overcomes both issues while it is still highly scalable with person and item ratings being updated after each response. In equilibrium, when neither learners nor items change, urnings are known to be binomially distributed variables, with the logits of the probability being the ability/difficulty in a Rasch model.

Urnings is a rating system where discrete parameters *u*_*p*_ and *u*_*i*_, the “urnings,” track the ability of a person and the difficulty of an item. Urnings assumes that the observed binary responses result from a game of chance played between persons and items matched-up with to probability *M*_*pi*_(*u*_*p*_, *u*_*i*_). The game proceeds with each player drawing a ball from an infinite urn containing red and green balls, the proportion of green balls being π_*p*_ in the person urn and π_*i*_ in the item urn. The game ends when the balls drawn are of different color and the player with the green ball wins. If the person wins, the item is solved and so the binary response corresponds to

$$X_{pi}=\left\{ \begin{array}{cc} 1 & \text{if}y_{p}^{*}=1\\ 0 & \text{if}y_{i}^{*}=1 \end{array}\right.$$

where $y_{p}^{*}$ and $y_{i}^{*}$ indicate whether the green ball was drawn by the person or the item. An easy derivation shows that the observed responses follow a Rasch model:

(8) $$\begin{array}{l} p(X_{pi}=1)=p(y_{p}^{*}=1,y_{i}^{*}=0|\theta_{p},\theta_{i})\\ \;=\frac{\pi_{p}(1-\pi_{i})}{\pi_{p}(1-\pi_{i})+(1-\pi_{p})\pi_{i}}=\frac{\exp(\theta_{p}-\theta_{i})}{1+\exp(\theta_{p}-\theta_{i})} \end{array}$$

where θ_*p*_ = ln(π_*p*_/(1−π_*p*_)) and similarly for θ_*i*_.

The urnings rating system mimics this game using finite sized urns. For each “real” game that is played, a corresponding simulated game is played with finite urns containing, respectively *u*_*p*_ and *u*_*i*_ green balls out of *n*^3^. Let *y*_*p*_ and *y*_*i*_ denote the outcome of the simulated game. If the result of the simulated game does not match that of the real game, the balls drawn are replaced with the outcome of the real game. If person *p* lost the simulated game but solved item *i*, the proportion of green balls for *p* is thus increased while the proportion of green balls for *i* is decreased. This can be summarized with the updated equations

(9) $$\begin{array}{r} u_{p}^{*}=u_{p}+y_{p}^{*}-y_{p} \end{array}$$

(10) $$\begin{array}{r} u_{i}^{*}=u_{i}+y_{i}^{*}-y_{i} \end{array}$$

where $u_{p}^{*}$ and $u_{i}^{*}$ are the proposed new configurations for the number of balls in each urn. This new configuration is then accepted or rejected using a Metropolis-Hastings acceptance probability to ensure that the ratings *u*_*p*_/*n* and *u*_*i*_/*n* converge to the proportions π_*p*_ and π_*i*_ when neither persons nor items change.

**Figure 3:**
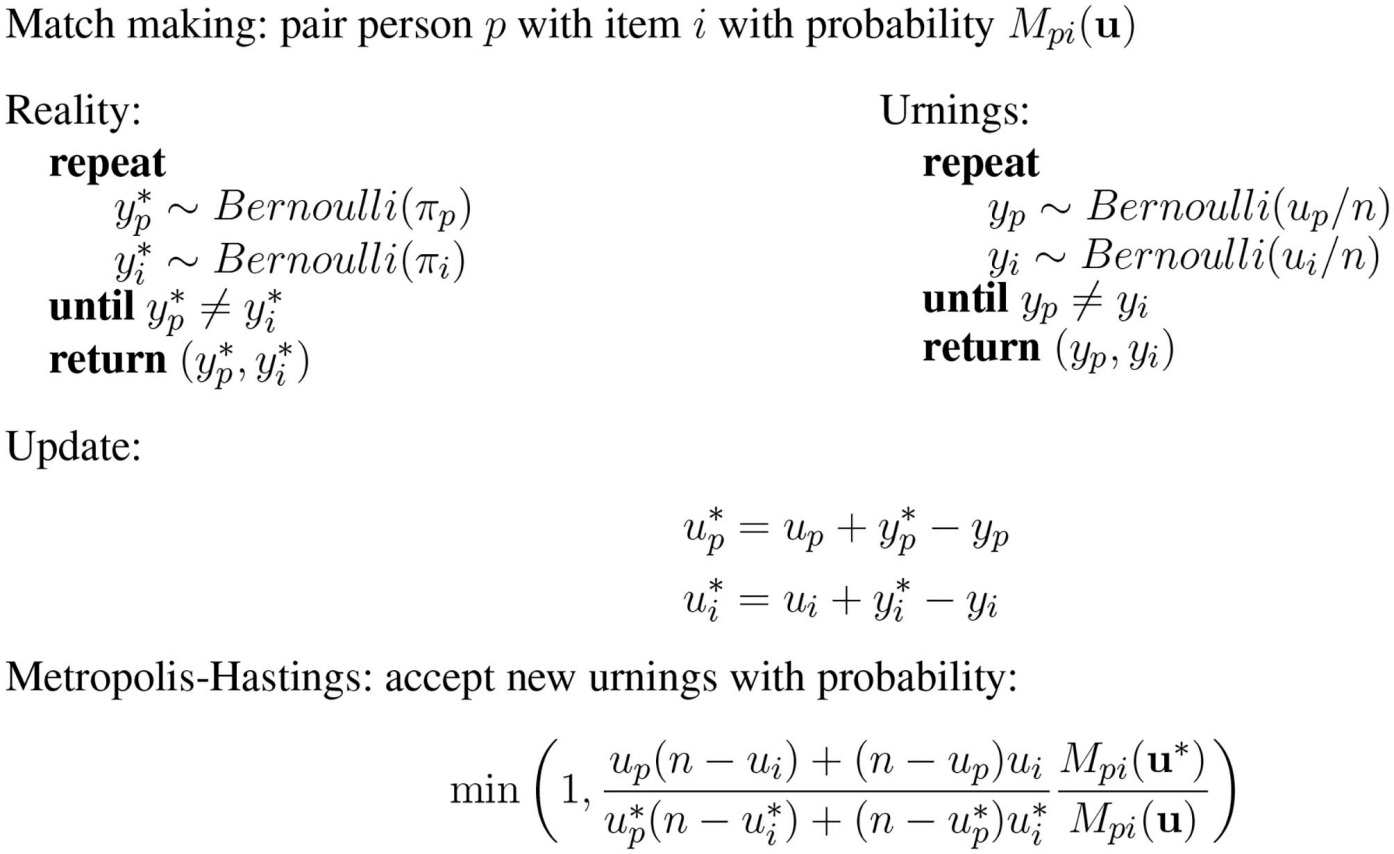
Urnings rating system.

Figure 3 gives an overview of the urnings updating scheme. Bolsinova et al. (2020) prove that each of the urn proportions forms a constructed Markov-chain such that the invariant distribution of $\text{u}={\left(u_{p},u_{i}\right)}^{⊺}$ is a binomial distribution with parameters *n* and $\pi ={\left(\pi_{p},\pi_{i}\right)}^{⊺}$. Note that the urn size *n* functions as a design parameter similar to the *K*-factor in Elo ratings. Larger urns mean that the system is more sensitive to change and the system converges more rapidly when the urns are smaller.

As the urnings rating system is designed to work with dichotomous response variables it is not directly applicable to the CR. However, through the use of the dyadic expansion, the continuous responses are transformed into a series of dichotomous responses. The urnings rating system can be applied directly to these dichotomous response variables that result from the dyadic expansion of the continuous responses. For a dyadic expansion of order *k*, we will use *k* urns for each person and *k* separate urns for each item. Due to the difference in discrimination, each person urn will be tracking $\theta_{p}/2^{j}$, where *j* ∈ {1,…,*k*} corresponds to the step in the dyadic expansion. Once the proportions in the urns are in equilibrium, one could combine them to get an overall estimate of θ_*p*_. This will be similar for the item urns and item difficulty. In the simulation section below, we show how this multi-urn solution can be used to identify model misspecification.

In the next section we derive an extension to the classical urnings rating system, which tracks the θ_*p*_ using a single urn.

### 3.2. Extension to Urnings

Recall that the *j*th item in the dyadic expansion corresponds to the ability $\theta_{p}/2^{j}$. We shall see that the differences in discrimination that derive from the dyadic expansion of the continuous response variables in the CR model translate into differences in the stakes of the game. The *stakes* of the urnings algorithm correspond to how much the number of green balls can increase (or decrease). In the classic urnings algorithm, the stakes are always equal to 1. In the extended urnings algorithm we allow items with different discriminations to combine. For a dyadic expansion of order *k* we let the item with the lowest discrimination, the final expansion, have a stake of one. For each previous item, we double the stakes such that the *j*th item in the dyadic expansion has a stake of 2^*k*−*j*^.

**Figure 4:**
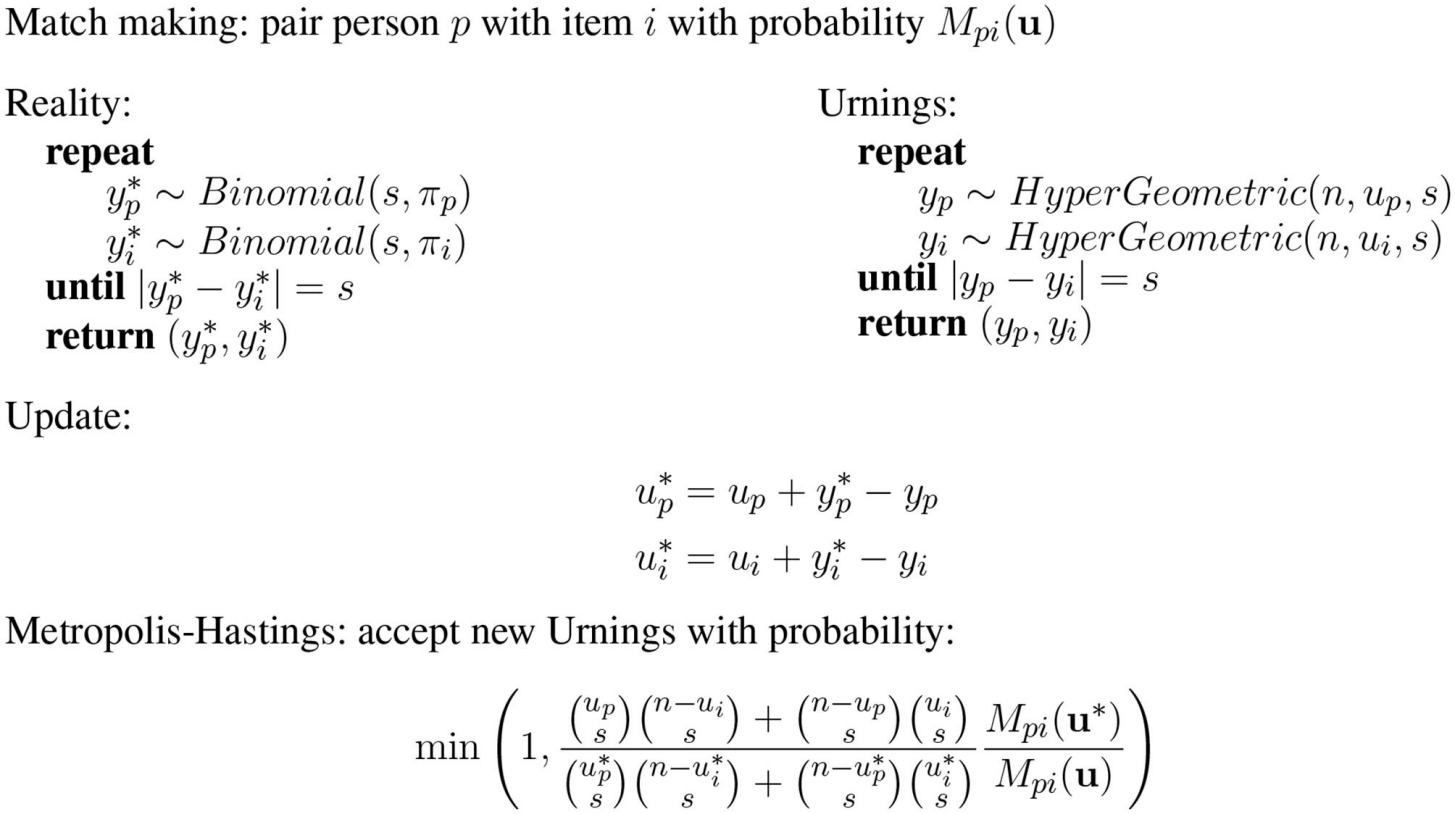
Extended Urnings rating system.

How does this impact the urnings update? Figure 4 has a summary of the extended urnings rating system. The observed binary outcomes *X*_*pi*_ are now assumed to be generated by the following game of chance. The game is same as above for classic urnings, except now the game has stakes *s*. For a game with stakes *s*, the process to generate the observed outcome is to continue drawing *s* balls from both urns ($y_{p}^{*}$ and $y_{i}^{*}$) until we get *s* green ones from the one urn and *s* red ones from the other. Thus

$$X_{pi}=\left\{ \begin{array}{cc} 1 & \text{if}y_{p}^{*}=s\\ 0 & \text{if}y_{i}^{*}=s \end{array}\right.$$

Similarly, a simulated game is played where balls are drawn (*y*_*p*_ and *y*_*i*_) from finite urns until *s* have been drawn from one urn and none from the other (without replacement). We once again just replace these *s* balls by *s* of the color consistent with the real item response. That is, a learner stands to lose or gain *s* balls based on her response to this particular item. This is why we refer to the discriminations as stakes in this context. Figure 4 has the updated Metropolis-Hastings acceptance probability, which is consistent with this extension. Theorem 1 provides the necessary theoretical justification for this correction. For a proof of the theorem see Appendix 1.

**THEOREM 1**. (Extension of Urnings Invariant Distribution) *If invariant distribution for the current configuration of balls is*

$$\begin{array}{l} p(u_{p},u_{i})={\left(\frac{s!}{n!/(n-s)!}\right)}^{2}\frac{\left(\begin{array}{c} u_{p}\\ s \end{array}\right)\left(\begin{array}{c} n-u_{i}\\ s \end{array}\right)+\left(\begin{array}{c} n-u_{p}\\ s \end{array}\right)\left(\begin{array}{c} u_{i}\\ s \end{array}\right)}{\pi_{p}^{s}{\left(1-\pi_{i}\right)}^{s}+{\left(1-\pi_{p}\right)}^{s}\pi_{i}^{s}}\left(\begin{array}{c} n\\ u_{p} \end{array}\right)\\ \;\pi_{p}^{u_{p}}{\left(1-\pi_{p}\right)}^{n-u_{p}}\left(\begin{array}{c} n\\ u_{i} \end{array}\right)\pi_{i}^{u_{i}}{\left(1-\pi_{i}\right)}^{n-u_{i}} \end{array}$$

*then the invariant distribution for the updated configuration of balls is the same, where s corresponds to the stakes*.

## 4. Simulation Study

We provide three simulation studies to illustrate the benefits of the proposed method. Simulation 1 shows how the urnings algorithm can recover the true ability of the persons and is robust to misspecification of the model generating the continuous responses. Simulation 2 simulates a more realistic setting and aims to show how our proposed approach handles the problems inherent in learning and assessment specified in the introduction. Simulation 3 highlights the problems inherent in any model which tracks ability and difficulty: these quantities are not separately identified, and it is easy to be misled when this is not taken into account (Bechger and Maris, 2015).

### 4.1. Simulation 1

We simulate 1,000 persons with ability uniformly distributed between -4 and 4, θ_*p*_ ~ *U*(−4, 4) and 100 items with difficulty distributed between −4 and 4, δ_*i*_ ~ *U*(−4, 4). We simulate a total of 100 million person-item interactions in order to create a data set that is comparable to the large-scale learning system data that the model is built for. At each interaction, a randomly sampled person and item is picked. The person's response is then simulated from the CR model based on their ability and the item's difficulty. This continuous response is then expanded using the dyadic expansion of order 3 to create three dichotomous responses. These dichotomous responses are then tracked by the multi-urn system with learner urns having an urn size of 50 and the item urns having urn sizes of 100.

#### 4.1.1. Tracking With Multiple Urns

**Figure 5:**
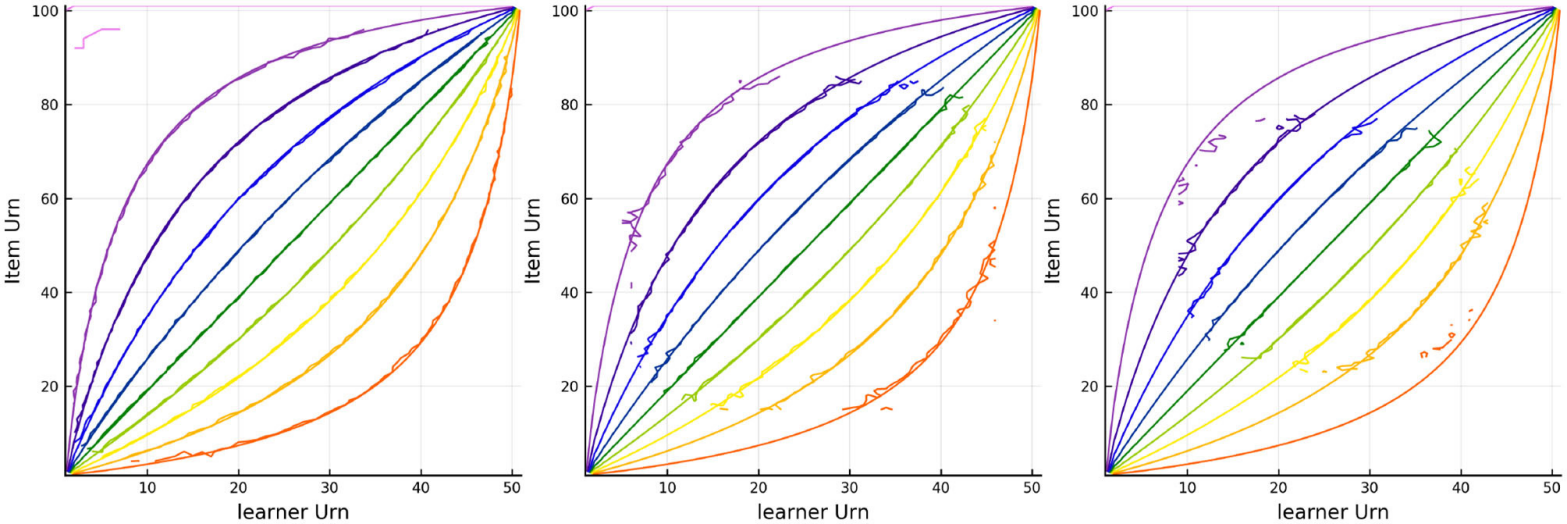
Contours for the predicted and observed proportion of correct responses for every combination of Urnings from simulation 1. Plots from left to right correspond to the urn associated with the respective step in the dyadic expansion.

**Figure 6:**
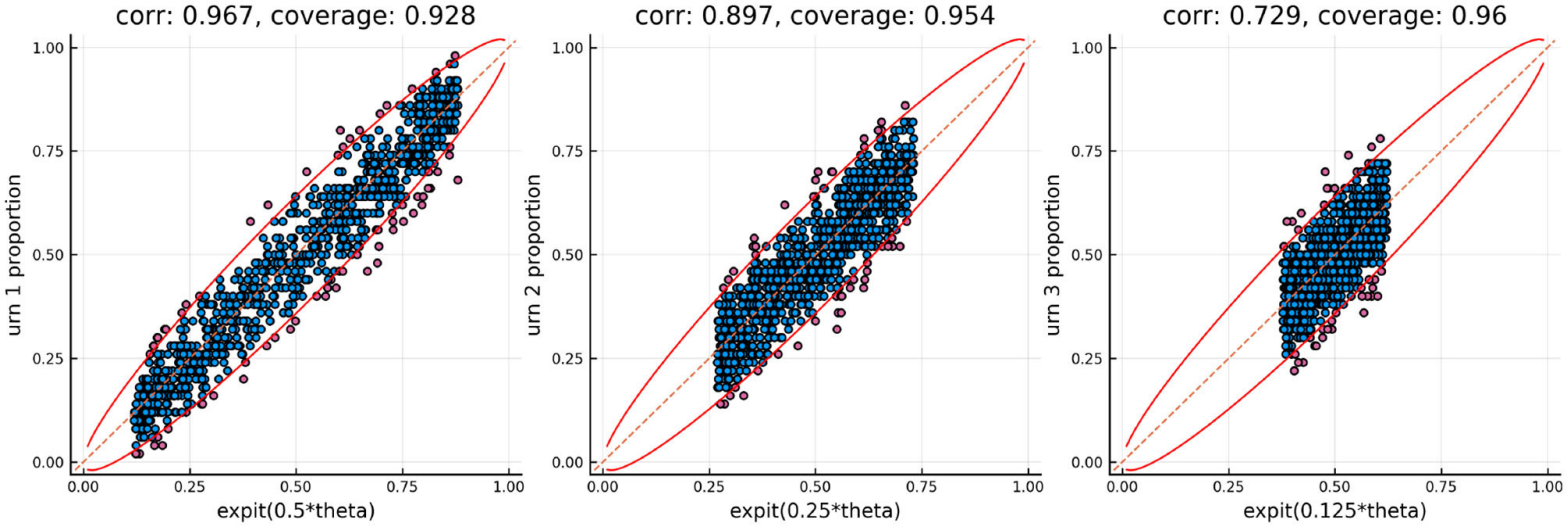
Urn proportions of the three urns plotted against the expit of the scaled ability, $\theta_{p}/2^{k}$ where *k* indexes the dyadic expansion. A 95% confidence interval is included along with the correlation and coverage.

The results of tracking the responses using the three urn system is in Figures 5, 6. The colored lines in Figure 5 correspond to the probability contours for the probability an item is answered correctly (from low probability given by purple to high probability given by red) given the urns for the person (horizontal axis) and the urns for the item (vertical axis). The smooth colored lines correspond to the expected probabilities while the noisier colored lines plotted on top correspond to the observed proportion of correct responses for every combination of Urnings from simulation 1. These plots show that there is good model fit, especially in the first urn. Figure 6 shows the final urn proportions in the three urns plotted against the simulated ability values (on the inverse logit scale, which we call “expit”). In red is the implied 95% confidence ellipse. The blue points are within the 95% ellipse while the red ones are outside of it. Each plot in Figure 6 also shows the correlation and the proportion of points within the ellipse (the coverage) in the plot title.

#### 4.1.2. Model Misspecification

How robust is this approach to deviations from the assumptions? We investigate this through simulating from a different underlying model. The learning and assessment system Math Garden also has continuous responses and assumes the same distribution for the scores as we have. The scores in Math Garden are derived as a particular function of response accuracy, i.e., was the response correct or incorrect, and response time to produce the continuous item score in such a way that penalizes fast incorrect responses. Specifically, *S*_*i*_ = (2*Y*_*i*_−1)(*d*−*T*_*i*_) where *Y*_*i*_ indicates whether the response was correct or not and *T*_*i*_ is time when the time-limit for responding is set to *d*. However, the fact that time is, literally, monetized in Math Garden, may entice learners to employ a different, more economic utility-based rule. Students may value their time and thus the relationship between their response scores, accuracy, and time may be *S*_*i*_ = *Y*_*i*_−*T*_*i*_ in which a slow incorrect response has a large negative score. The question is can we detect that learners follow the alternative scoring rule rather than the intended one? The answer is yes. We will show this by means of a simulation.

We augment the first simulation. Rather than simulating from the CR model we will simulate from the distribution implied by the scoring rule *S*_*i*_ = *Y*_*i*_−*T*_*i*_. One can show that in order to simulate from this distribution we can do the following. We first simulate the response *Y*_*i*_ from the CR model, but if the response is <0.5, *Y*_*i*_ < 0.5, then we set the score to be *Y*_*i*_ = 0.5−*Y*_*i*_. One of the benefits of using three separate urns to track the ability is that model misfit can be detected by comparing the urns to each other. The relationship between the true urn proportions is a known function. Specifically, if θ_*p*_ are the true simulated abilities we can plot the inverse logit of θ_*p*_/2 against the inverse logit of θ_*p*_/4. If the observed own proportions don't follow this relationship there is model misfit.

**Figure 7:**
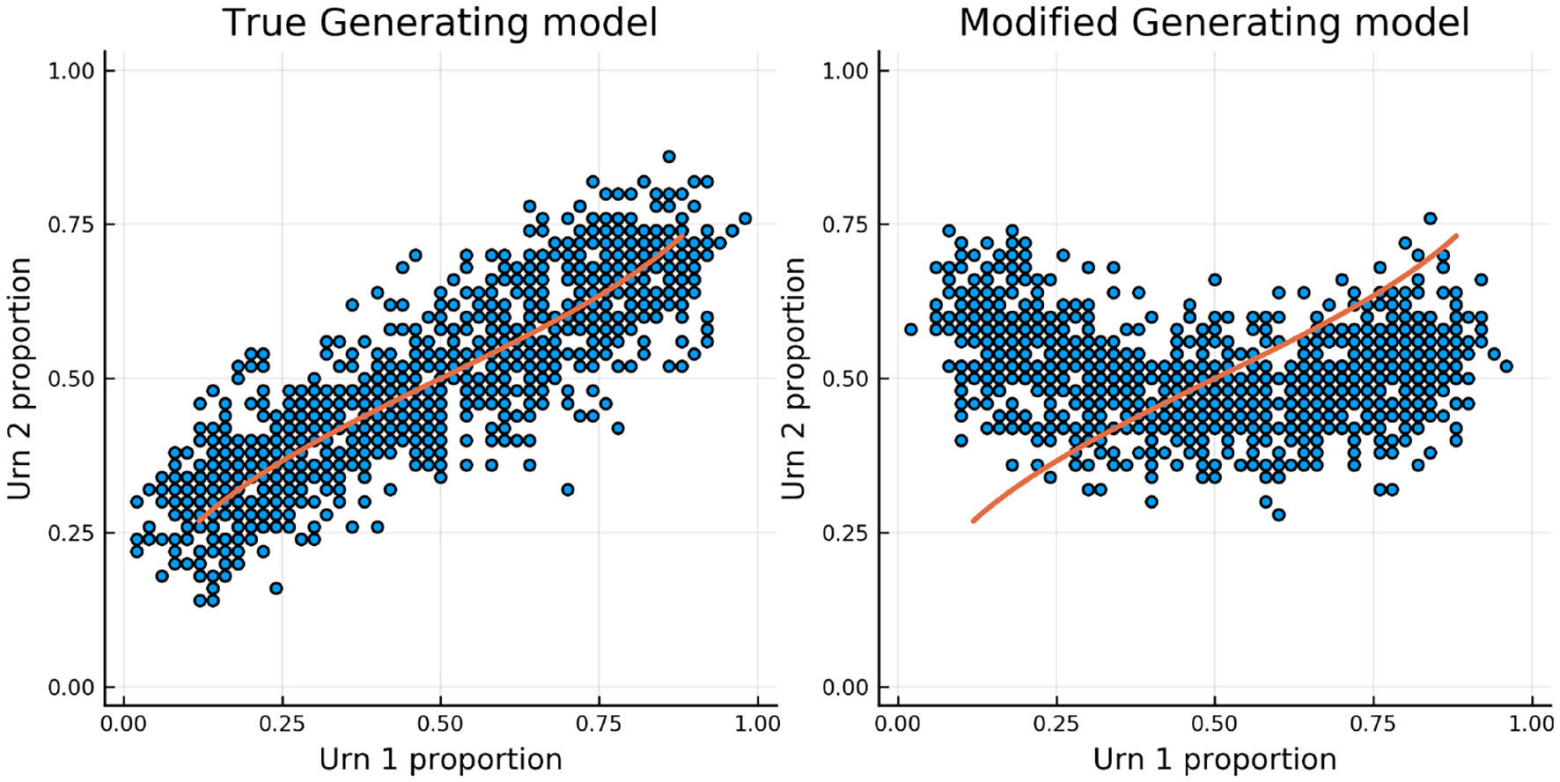
Urn proportions in urn 1 plotted against urn proportions in urn 2 using the true generating model and the alternative model.

Figure 7 shows the relationship between the urn proportions in urns 1 and 2 using the true generating model and the modified generating model. This figure shows that when the generating model is the modified one the model misspecification can be detected as the relationship between the urn proportions follows a U-shaped curve rather than the expected monotonic relationship.

### 4.2. Simulation 2

For Simulation 2 we consider a more realistic setting. Specifically, we deal with two problems in learning and assessment systems: *the problem of change* and *the problem of personalization and adaptation*. We allow the ability of the persons to change over time. Specifically, the ability changes as a function of time according to a generalized logistic function

(11) $$\begin{array}{r} \theta_{p}\left(t\right)=\theta_{p1}+\frac{\theta_{p2}-\theta_{p1}}{1+\exp\left(-\alpha_{p}t\right)} \end{array}$$

where *t* is the simulation index (from 1 to 10^8^) mapped to the interval (−4,4), θ_*p*1_ ~ U(−4, 4), θ_*p*2_ ~ U(−4, 4), and α_*p*_ ~ Gamma(1, 1). The item difficulty is simulated from the uniform again, δ_*i*_ ~ U(−4, 4) and held constant. Once again, we simulate 10^8^ responses from the continuous Rasch model where a person is (uniformly) randomly selected but now a random item is selected by choosing one with the following weights

(12) $$\begin{array}{r} M_{pi}\left(\text{u}\right)=\exp(-2(\ln(u_{p}+1)/(n_{p}-u_{p}+1))\\ \;-\ln\left(u_{i}+1\right)/\left(n_{i}-u_{i}+1\right){)}^{2} \end{array}$$

where *u*_*p*_ corresponds to the selected person's urn proportion, *u*_*i*_ corresponds to item *i*'s urn proportion, and *n*_*p*_ and *n*_*i*_ the person and item urn sizes, respectively. This results in items whose difficulty are closer to the selected person's ability being more likely selected. For this simulation we track the ability using a single urn with urn sizes of 420 for both the person and item urns.

**Figure 8:**
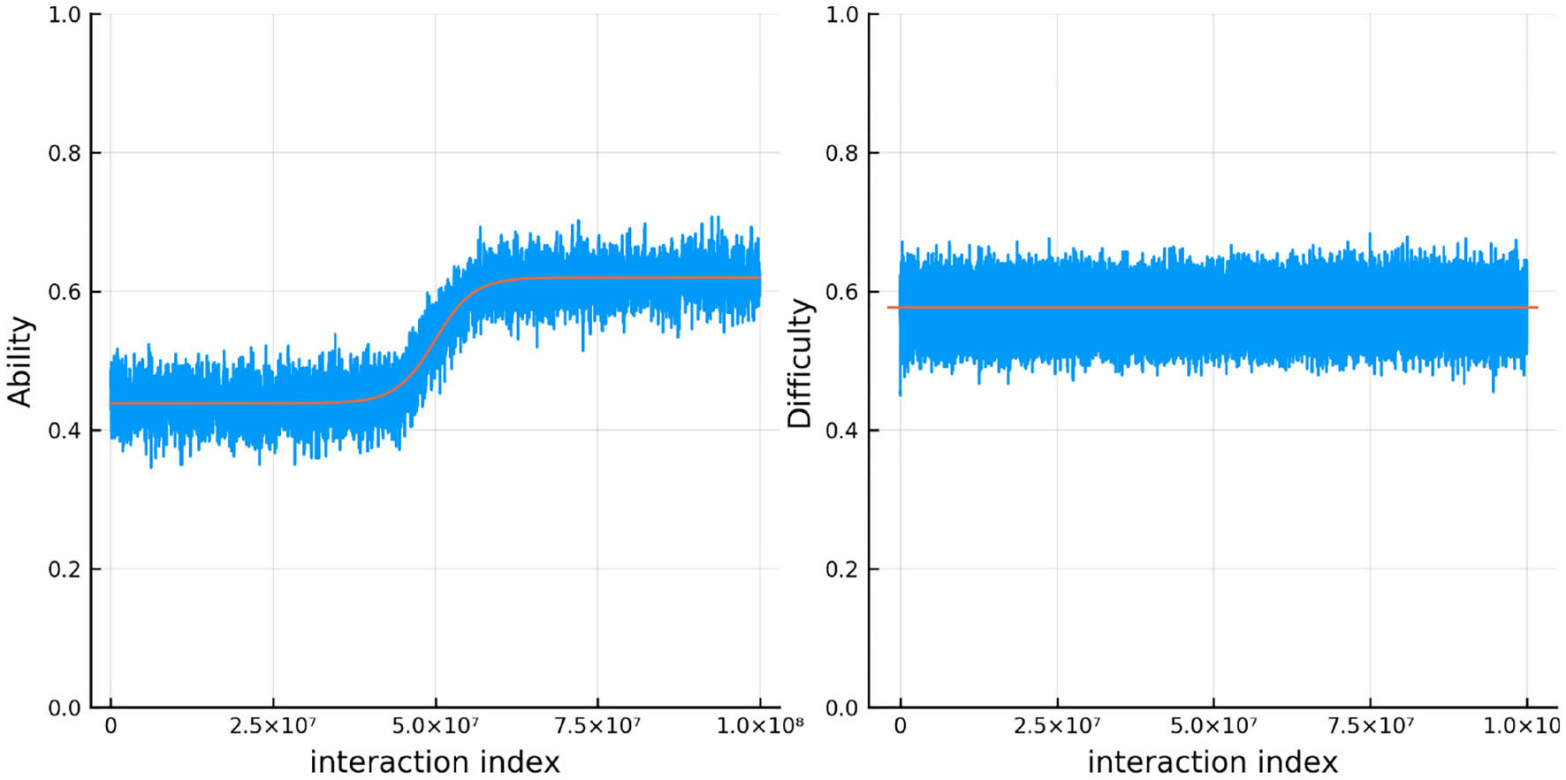
The true (solid red line) and estimated (blue line) change in ability (left) for 1 specific person and item difficulty (right) for 1 specific item in simulation 2.

**Figure 9:**
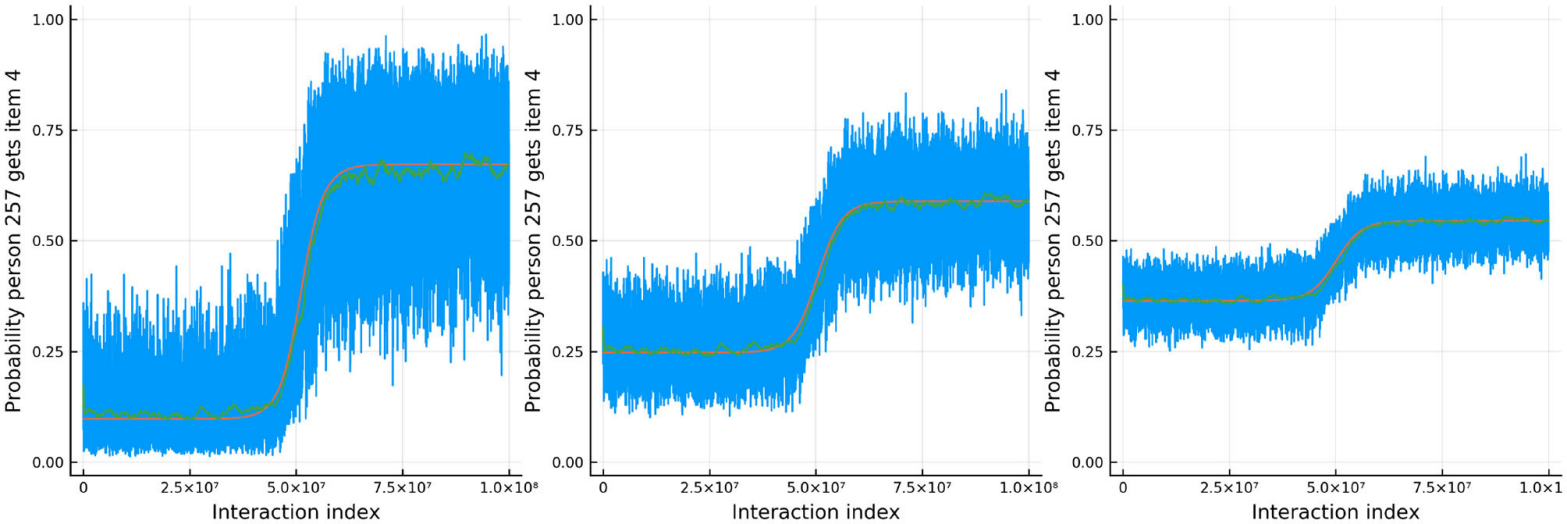
The probability that a specific person answers the *d*th item in the dyadic expansion of a specific item correctly in simulation 2.

Figure 8 shows the results for one person and one item in particular. In red is the true ability and difficulty of this person and item and the blue trace line is the urn proportion. These show that the extended Urnings rating system can track the change in ability well. We can increase the urn size if we wish to decrease the variance in the urn proportions. Another traceplot that can be generated is Figure 9. The leftmost plot in this figure is the probability that the response to the first dyadic expansion of a particular item is 1, the middle one is the 2nd dyadic expansion of the same person and item, and the rightmost plot is the third expansion. This also shows good fit to the simulated data. Along with increasing the urn size in order to decrease variance we can also keep track of a running mean. In Figure 9 we also plot the average of the previous 2,000 probabilities at each new interaction which closely tracks the true probability.

### 4.3. Simulation 3

For the final simulation we explore the trouble with every measurement model, which relates ability to difficulty as the Rasch model does: the issue of unidentifiability of these parameters. In most assessment frameworks this issue is often circumvented by several assumptions, such as the assumption that the abilities of the persons and the difficulties of the items are static and not changing. Additionally, some arbitrary zero point must be decided on, which is typically that the average difficulty of the population of items is equal to zero. In this final simulation, we challenge some of these assumptions as typically happens in real data, especially in learning systems.

**Figure 10:**
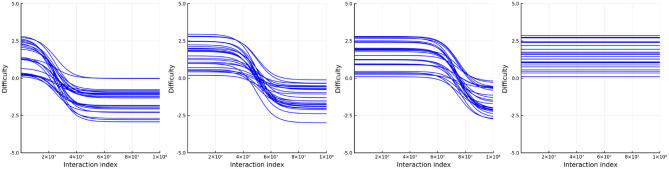
True item difficulties in simulation 3.

As before, we allow the ability to change over time in the same was as we did in simulation 2. However, we restrict the change in ability to only be positive by sampling θ_*p*1_ ~ U(−4, 0) and θ_*p*2_ ~ U(0, 4) so that each person's ability increases. Furthermore, we allow the difficulty of the items to change over time. The item difficulties change in the same way as the person ability, but they all decrease over time. Specifically, the difficulty is

(13) $$\begin{array}{r} \delta_{i}\left(t\right)=\delta_{i1}+\frac{\delta_{i2}-\delta_{i1}}{1+\exp\left(-2\left(t-t_{0}\right)\right)} \end{array}$$

where δ_*i*1_ ~ U(0, 4) and δ_*i*2_ ~ U(−4, 0). Additionally, we split the items into four groups such that the point, *t*_0_ (at which the difficulty is half way between its starting difficulty, δ_1_, to its ending difficulty, δ_*i*2_) varies between groups. In the first group of items the mid-point is at the first quarter of the number of simulated interactions, the second group is half way through the simulated interactions (just like the person ability), the third group is three quarters of the way through the simulated interactions, and the last group does not change in ability. Figure 10 plots the (true) change in item difficulty over the simulated interactions. In this way we simulate an experience that is close to a learning environment. Items whose relative difficulty decreases early on represent items related to skills which the persons learn early on in the learning environment. Just as in simulation 2, at each interaction we randomly pick a person and then select an item using the same weights as described in simulation 2. The single urn scheme is used to track the abilities and difficulties with urns of size 420 for both persons and items.

**Figure 11:**
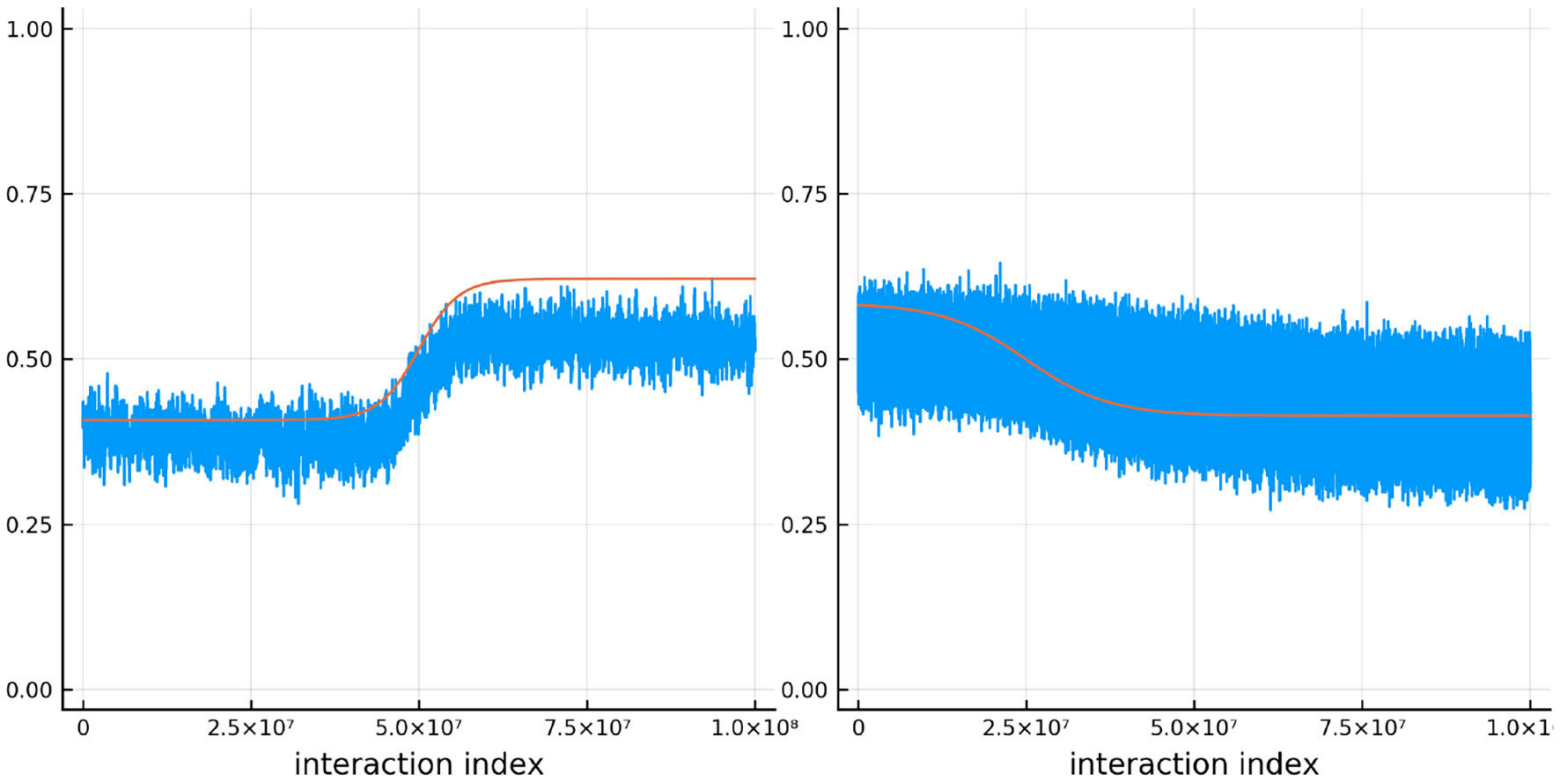
The true (solid red line) and estimated (blue line) change in ability (left) for one specific person and item difficulty (right) for one specific item in simulation 3.

**Figure 12:**
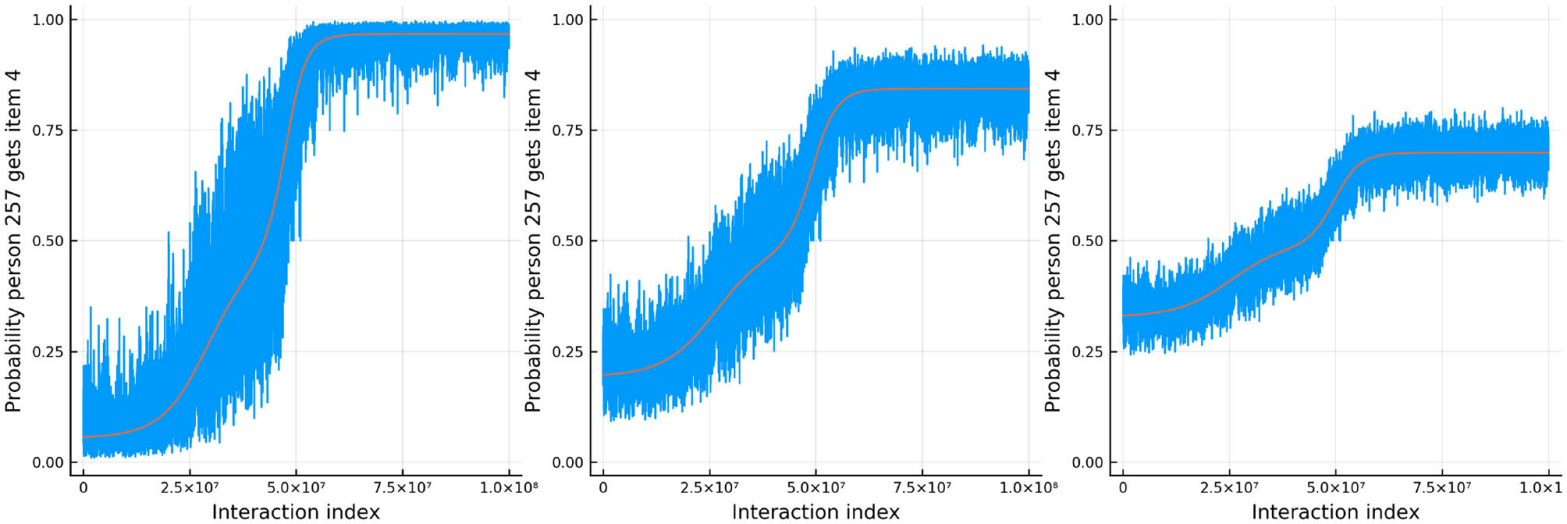
The probability that a specific person answers the *d*th item in the dyadic expansion of a specific item correctly in simulation 3.

Figure 11 shows the true and estimated ability and difficulty for a particular person and a particular item. The true ability change is in red on the left and the true difficulty is in red on the right. In blue, the urn proportion for the ability on the left and the difficulty on the right. What is happening here? Clearly the urn proportions do not track the true values; this is most evident with the ability on the left. As the number of balls in the person and item urns is always fixed, if we allow the items to become easier over time and the person abilities to increase over time, the persons are literally stealing balls away from the items. This results in under-estimation of the person abilities and over-estimation of the item difficulties. In the previous simulation this effect was circumvented by allowing the distribution of ability (and difficulty) to be the same at the start of the simulation and at the end, by allowing some people's ability to increase and others to decrease (and the item difficulty was kept constant). This is not the case in this simulation. Only quantities that are properly contextualized can be accurately tracked, such as the probability that a person answers an item correctly. Consider Figure 12. As in the previous simulation, this figure plots the probability that a particular person gets one of the dyadic expansion items correct on a particular item.

## 5. Discussion

In this article, we have proposed a new method to analyze data generated by massive online learning systems, such as DET or Math Garden, based on the CR model and the Urnings ratings system. We have demonstrated its feasibility using simulation.

The approach described here is new and based on three ingredients. First, we found that the SRT model is a special case of a Rasch model for continuous item responses. Second, we established that, if the CR model holds, continuous responses can be transformed to independent binary responses that follow the Rasch model and contain most of the information in the original responses. Of course, the Rasch model is known to not always fit the data, as it assumes each item discriminates equally well (Verhelst, 2019). We have discussed the topic of model misspecification (with regard to the misspecification of the scoring rule rather than the true data-generating process), but the focus of this paper has been on the use of the CR in the context of a learning system. Third, the urnings rating system can be applied to the binary responses to track both learners and items in real time.

In the introduction, three unique problems with large-scale, high-stakes, online, anywhere anytime learning and testing were identified. Having dealt with the problem of change and of personalization and adaptation we now briefly comment on the cold start problem. Having introduced the notion of stakes, as a way of dealing with differences in item discrimination, we can reuse the same idea for addressing the cold start problem. When a new person or item is added, we initially multiply their stakes by some number. This has the effect, similar to decreasing the urn size, of taking large(r) steps, and hence more rapidly converging to the “correct” value, but with a larger standard error. After some initial responses have been processed, the multiplier can decrease to one. Note that, in principle, the same approach can be used continuously to adjust the stakes depending on how fast or slow a person or item parameter is changing.

An extension of the urnings system was introduced in order to make use of the dichotomous responses with varying discriminations. It will be clear that we have only begun to explore the possibilities offered by the new method.

## Data Availability Statement

The datasets generated for this study are available on request to the corresponding author.

## Author Contributions

GM developed the initial idea. BD, MB, TB, and GM were involved in further developments, writing, and critical revisions. BD and GM developed code and simulations. All authors contributed to the article and approved the submitted version.

## Conflict of Interest

BD, TB, and GM work at ACT, Inc. The remaining author declares that the research was conducted in the absence of any commercial or financial relationships that could be construed as a potential conflict of interest.

## Footnotes

^1^After re-scaling, if *X* ~ SRT(η) then $Y=\frac{1}{2}\left(X-1\right)\sim \text{CR}\left(2\eta \right)$.

^2^The infinite sum $\frac{1}{4}+\frac{1}{16}+\frac{1}{64}+\ldots \;$ is equal to $\frac{1}{3}$.

^3^Note that in practice the number of balls in the person urns and item urns don't have to be equal, but for notations sake we will keep them the same.
