# Supplementary material for: A Rasch Model and Rating System for Continuous Responses Collected in Large-Scale Learning Systems
Source: Front Psychol. 2020 Dec 18;11:500039. doi: 10.3389/fpsyg.2020.500039 (PMC7775507; doi:10.3389/fpsyg.2020.500039)
Supplement: Supplementary file 1 [file Presentation_1.pdf]

# A Proof of Theorem 1

**Theorem 1** (Extension of Urnings Invariant Distribution)**.** *If invariant distribution for the current configuration of balls is*

$$p(u_p, u_i) = \left(\frac{s!}{n!/(n-s)!}\right)^2 \frac{\binom{u_p}{s}\binom{n-u_i}{s} + \binom{n-u_p}{s}\binom{u_i}{s}}{\pi_p^s(1-\pi_i)^s + (1-\pi_p)^s\pi_i^s} \binom{n}{u_p}\pi_p^{u_p}(1-\pi_p)^{n-u_p}\binom{n}{u_i}\pi_i^{u_i}(1-\pi_i)^{n-u_i}$$

*then the invariant distribution for the updated configuration of balls is the same, where $s$ corresponds to the stakes.*

*Proof.* Let $\tilde{p}(u_p, u_i)$ be the distribution of the updated configuration. We will show this is equal to $p(u_p, u_i)$. There are 4 distinct ways to obtain an updated configuration of $(u_p, u_i)$. These 4 ways vary in the current configuration, the observed outcome, and the simulated outcome:

1. $(u_p - s, u_i + s)$ and $(y_p^* = s, y_p = 0)$
2. $(u_p, u_i)$ and $(y_p^* = 0, y_p = 0)$
3. $(u_p, u_i)$ and $(y_p^* = s, y_p = s)$
4. $(u_p + s, u_i - s)$ and $(y_p^* = 0, y_p = s)$

By the law of total probability we have

$$\begin{aligned}\tilde{p}(u_p, u_i) = &\underbrace{p(y_p^* = s)p(y_p = 0|u_p - s, u_i + s)p(u_p - s, u_i + s)}_{A} + \\ &\underbrace{p(y_p^* = 0)p(y_p = 0|u_p, u_i)p(u_p, u_i)}_{B} + \\ &\underbrace{p(y_p^* = s)p(y_p = s|u_p, u_i)p(u_p, u_i)}_{C} + \\ &\underbrace{p(y_p^* = 0)p(y_p = s|u_p + s, u_i - s)p(u_p + s, u_i - s)}_{D}\end{aligned} \tag{14}$$

where

$$A = \frac{\pi_p^s(1-\pi_i)^s}{\pi_p^s(1-\pi_i)^s + \pi_i^s(1-\pi_p)^s} \frac{\binom{u_i+s}{s}\binom{n-(u_p-s)}{s}}{\binom{u_i+s}{s}\binom{n-(u_p-s)}{s} + \binom{u_p-s}{s}\binom{n-(u_i+s)}{s}} p(u_p - s, u_i + s) \tag{15}$$

$$B = \frac{\pi_i^s(1-\pi_p)^s}{\pi_p^s(1-\pi_i)^s + \pi_i^s(1-\pi_p)^s} \frac{\binom{u_i}{s}\binom{n-u_p}{s}}{\binom{u_i}{s}\binom{n-u_p}{s} + \binom{u_p}{s}\binom{n-u_i}{s}} p(u_p, u_i) \tag{16}$$

$$C = \frac{\pi_p^s(1-\pi_i)^s}{\pi_p^s(1-\pi_i)^s + \pi_i^s(1-\pi_p)^s} \frac{\binom{u_p}{s}\binom{n-u_i}{s}}{\binom{u_i}{s}\binom{n-u_p}{s} + \binom{u_p}{s}\binom{n-u_i}{s}} p(u_p, u_i) \tag{17}$$

$$D = \frac{\pi_i^s(1-\pi_p)^s}{\pi_p^s(1-\pi_i)^s + \pi_i^s(1-\pi_p)^s} \frac{\binom{u_p+s}{s}\binom{n-(u_i-s)}{s}}{\binom{u_i-s}{s}\binom{n-(u_p+s)}{s} + \binom{u_p+s}{s}\binom{n-(u_i-s)}{s}} p(u_p + s, u_i - s) \tag{18}$$

Next note that using the following binomial coefficient identities

$$\binom{n}{k-s} = \frac{\binom{k}{s}}{\binom{n-(k-s)}{s}}\binom{n}{k} \qquad \binom{n}{k+s} = \frac{\binom{n-k}{s}}{\binom{k+s}{s}}\binom{n}{k}$$

we can make the following equality

$$
\begin{aligned}
p(u_p - s, u_i + s) = {} & \left(\frac{s!}{n!/(n-s)!}\right)^2 \frac{\binom{u_p - s}{s}\binom{n-(u_i+s)}{s} + \binom{n-(u_p-s)}{s}\binom{u_i+s}{s}}{\pi_p^s(1-\pi_i)^s + (1-\pi_p)^s\pi_i^s} \times \\
& \binom{n}{u_p - s}\pi_p^{u_p - s}(1-\pi_p)^{n-(u_p-s)}\binom{n}{u_i + s}\pi_i^{u_i+s}(1-\pi_i)^{n-(u_i+s)} \qquad (19) \\
= {} & \left(\frac{s!}{n!/(n-s)!}\right)^2 \frac{\binom{u_p - s}{s}\binom{n-(u_i+s)}{s} + \binom{n-(u_p-s)}{s}\binom{u_i+s}{s}}{\pi_p^s(1-\pi_i)^s + (1-\pi_p)^s\pi_i^s} \times \\
& \frac{\pi_i^s(1-\pi_p)^s}{\pi_p^s(1-\pi_i)^s}\frac{\binom{u_p}{s}\binom{n-u_i}{s}}{\binom{u_i+s}{s}\binom{n-(u_p-s)}{s}}\binom{n}{u_p}\pi_p^{u_p}(1-\pi_p)^{n-u_p}\binom{n}{u_i}\pi_i^{u_i}(1-\pi_i)^{n-u_i} \qquad (20) \\
= {} & \frac{\binom{u_p - s}{s}\binom{n-(u_i+s)}{s} + \binom{n-(u_p-s)}{s}\binom{u_i+s}{s}}{\binom{u_p}{s}\binom{n-u_i}{s} + \binom{u_i}{s}\binom{n-u_p}{s}}\frac{\pi_i^s(1-\pi_p)^s}{\pi_p^s(1-\pi_i)^s}\frac{\binom{u_p}{s}\binom{n-u_i}{s}}{\binom{u_i+s}{s}\binom{n-(u_p-s)}{s}}p(u_p, u_i) \qquad (21)
\end{aligned}
$$

Using the previous equality we can rewrite $A$ as

$$
A = \frac{\pi_i^s(1-\pi_p)^s}{\pi_p^s(1-\pi_i)^s + \pi_i^s(1-\pi_p)^s}\frac{\binom{u_p}{s}\binom{n-u_i}{s}}{\binom{u_i}{s}\binom{n-u_p}{s} + \binom{u_p}{s}\binom{n-u_i}{s}}p(u_p, u_i) \qquad (22)
$$

This allows us to combine $A$ and $B$

$$
A + B = \frac{\pi_i^s(1-\pi_p)^s}{\pi_p^s(1-\pi_i)^s + \pi_i^s(1-\pi_p)^s}p(u_p, u_i) \qquad (23)
$$

Similarly, we can obtain an equality for $p(u_p + s, u_i - s)$ as we did for $p(u_p - s, u_i + s)$ above

$$
\begin{aligned}
p(u_p + s, u_i - s) = {} & \left(\frac{s!}{n!/(n-s)!}\right)^2 \frac{\binom{u_p + s}{s}\binom{n-(u_i-s)}{s} + \binom{n-(u_p+s)}{s}\binom{u_i-s}{s}}{\pi_p^s(1-\pi_i)^s + (1-\pi_p)^s\pi_i^s} \times \\
& \binom{n}{u_p + s}\pi_p^{u_p + s}(1-\pi_p)^{n-(u_p+s)}\binom{n}{u_i - s}\pi_i^{u_i-s}(1-\pi_i)^{n-(u_i-s)} \qquad (24) \\
= {} & \left(\frac{s!}{n!/(n-s)!}\right)^2 \frac{\binom{u_p + s}{s}\binom{n-(u_i-s)}{s} + \binom{n-(u_p+s)}{s}\binom{u_i-s}{s}}{\pi_p^s(1-\pi_i)^s + (1-\pi_p)^s\pi_i^s} \times \\
& \frac{\pi_i^s(1-\pi_p)^s}{\pi_p^s(1-\pi_i)^s}\frac{\binom{u_p}{s}\binom{n-u_i}{s}}{\binom{u_i-s}{s}\binom{n-(u_p+s)}{s}}\binom{n}{u_p}\pi_p^{u_p}(1-\pi_p)^{n-u_p}\binom{n}{u_i}\pi_i^{u_i}(1-\pi_i)^{n-u_i} \qquad (25) \\
= {} & \frac{\binom{u_p + s}{s}\binom{n-(u_i-s)}{s} + \binom{n-(u_p+s)}{s}\binom{u_i-s}{s}}{\binom{u_p}{s}\binom{n-u_i}{s} + \binom{u_i}{s}\binom{n-u_p}{s}}\frac{\pi_i^s(1-\pi_p)^s}{\pi_p^s(1-\pi_i)^s}\frac{\binom{u_p}{s}\binom{n-u_i}{s}}{\binom{u_i-s}{s}\binom{n-(u_p+s)}{s}}p(u_p, u_i) \qquad (26)
\end{aligned}
$$

This allows us to rewrite $D$ as

$$
D = \frac{\pi_p^s(1-\pi_i)^s}{\pi_p^s(1-\pi_i)^s + \pi_i^s(1-\pi_p)^s}\frac{\binom{u_i}{s}\binom{n-u_p}{s}}{\binom{u_p}{s}\binom{n-u_i}{s} + \binom{u_i}{n-u_p}s}p(u_p, u_i) \qquad (27)
$$

which allows us to combine $C$ and $D$

$$
C + D = \frac{\pi_p^s(1-\pi_i)^s}{\pi_p^s(1-\pi_i)^s + \pi_i^s(1-\pi_p)^s}p(u_p, u_i) \qquad (28)
$$

and finally we combine all 4 terms

$$A + B + C + D = p(u_p, u_i) \tag{29}$$

□
